# Assessment of the prognostic role of a 94-single nucleotide polymorphisms risk score in early breast cancer in the SIGNAL/PHARE prospective cohort: no correlation with clinico-pathological characteristics and outcomes

**DOI:** 10.1186/s13058-017-0888-4

**Published:** 2017-08-22

**Authors:** Elsa Curtit, Xavier Pivot, Julie Henriques, Sophie Paget-Bailly, Pierre Fumoleau, Maria Rios, Hervé Bonnefoi, Thomas Bachelot, Patrick Soulié, Christelle Jouannaud, Hugues Bourgeois, Thierry Petit, Isabelle Tennevet, David Assouline, Marie-Christine Mathieu, Jean-Philippe Jacquin, Sandrine Lavau-Denes, Ariane Darut-Jouve, Jean-Marc Ferrero, Carole Tarpin, Christelle Lévy, Valérie Delecroix, Véronique Trillet-Lenoir, Oana Cojocarasu, Jérôme Meunier, Jean-Yves Pierga, Pierre Kerbrat, Céline Faure-Mercier, Hélène Blanché, Mourad Sahbatou, Anne Boland, Delphine Bacq, Céline Besse, Gilles Thomas, Jean-François Deleuze, Iris Pauporté, Gilles Romieu, David G. Cox

**Affiliations:** 1 Hôpital Jean-Minjoz, Centre Hospitalier Universitaire, UMR 1098 INSERM-EFS-Université de Bourgogne Franche-Comté, Boulevard Fleming, 25000 Besançon, France; 2 Centre Hospitalier Universitaire, Unité de Méthodologie et de Qualité de Vie en Cancérologie, 2 place St Jacques, 25000 Besançon, France; 3 Georges-François Leclerc, 1 Rue du Professeur Marion, 21000 Dijon, France; 4 Institut de Cancérologie de Lorraine - Alexis Vautrin, département d’Oncologie Médicale, 6, avenue de Bourgogne, 54511 Vandoeuvre Les Nancy Cedex, France; 5 Institut Bergonié, Département d’Oncologie Médicale, 229 Cours de l’Argonne, 33000 Bordeaux, France; 6 Centre Léon Bérard, Département de Cancérologie Médicale, 28 rue Laënnec, Lyon Cedex 08, France; 7 Institut de Cancérologie de l’Ouest, Service Oncologie Médicale, 2 rue Moll, 49993 Angers Cedex 09, France; 8 Institut Jean Godinot, Service Oncologie Médicale, 1 rue du Général Koenig, 51056 Reims cedex, France; 9 Clinique Victor Hugo-Centre Jean Bernard, 18 rue Victor Hugo, 72015 Le Mans Cedex 2, France; 10 Centre Paul Strauss, Service d’Oncologie Médicale, 3 rue de la Porte de l’Hôpital, 67065 Strasbourg Cedex, France; 11 Centre Henri Becquerel, rue d’Amiens, 76038 Rouen, France; 12 Institut Daniel Hollard, Service Oncologie Médicale, 8 rue du Docteur Calmette, 38028 Grenoble Cedex 01, France; 13 Institut Gustave Roussy, Comité de Pathologie mammaire, 39 rue Camille Desmoulins, 94805 Villejuif Cedex, France; 14 Institut de Cancérologie Lucien Neuwirth, Service Oncologie Médicale, 108 bis avenue Albert Raimond, 42270 Saint Priest en Jarez, France; 15 Centre Hospitalier de Limoges, Service d’Oncologie Médicale, 2 avenue Martin Luther King, 87042 Limoges Cedex, France; 16 Clinique Drévon, Centre d’oncologie et de radiothérapie du Parc, 18 cours du général de Gaulle, 21000 Dijon, France; 17 Centre Antoine Lacassagne, Département Oncologie Médicale, 33 avenue de Valombrose, 06189 Nice Cedex 02, France; 18 Institut Paoli-Calmettes, Département d’Oncologie Médicale, 232 Boulevard de Sainte-Marguerite, 13009 Marseille, France; 19 Centre François Baclesse, 3 avenue du Général Harris, 14076 Caen Cedex 5, France; 20 Centre Etienne Dolet, Pôle Mutualiste, Service Oncologie Médicale, 11 boulevard Georges Charpak, 44606 Saint Nazaire, France; 21 Centre Hospitalier Lyon Sud, Service d’Oncologie Médicale, 165 chemin du Grand Revoyet, 69495 Pierre-Benite Cedex, France; 22 Centre Hospitalier Le Mans, Service d’Onco-Hématologie et Médecine interne, 194 avenue Rubillard, 72037 Le Mans Cedex, France; 23 Centre Hospitalier Régional d’Orléans, Service d’Oncologie médicale, 1 rue Porte Madeleine, 45032 Orleans Cedex 1, France; 24 Institut Curie, Department of Medical Oncology, 26 rue d’Ulm, 75248 Paris Cedex 05, France; 25 Centre Eugène Marquis, Service Oncologie médicale, Rue de la Bataille Flandres-Dunkerque, CS 44229, 35042 Rennes Cedex, France; 26 Institut National du Cancer, Direction de la Recherche, 52 avenue Morizet, 92513 Boulogne-Billancourt, France; 27 Centre d’Etudes du Polymorphisme Humain, 27 rue Juliette Dodu, 75010 Paris, France; 28 Centre National du Génotypage, 2 rue Gaston Crémieux, CP 5721, 91057 Evry Cedex, France; 29 Synergie Lyon Cancer, Centre Léon Bérard, 28 rue Laënnec, Lyon Cedex 08, France; 30 Oncologie Sénologie, ICM Institut Régional du Cancer, 34298 Montpellier Cedex, France; 31 Centre de Recherche en Cancérologie de Lyon, INSERM U1052 - Centre Léon Bérard, 28 rue Laennec, 69373 Lyon, France; 32 Department of Medical Oncology, University Hospital Jean Minjoz, 3, boulevard Alexandre Fleming, 25030 Besancon Cedex, France

**Keywords:** Breast cancer, Genetic variant, Single nucleotide polymorphism, Risk score, Prognosis

## Abstract

**Background:**

Genome-wide association studies (GWAS) have to date identified 94 genetic variants (single nucleotide polymorphisms (SNPs)) associated with risk of developing breast cancer. A score based on the combined effect of the 94 risk alleles can be calculated to measure the global risk of breast cancer. We aimed to test the hypothesis that the 94-SNP-based risk score is associated with clinico-pathological characteristics, breast cancer subtypes and outcomes in early breast cancer.

**Methods:**

A 94-SNP risk score was calculated in 8703 patients in the PHARE and SIGNAL prospective case cohorts. This score is the total number of inherited risk alleles based on 94 selected SNPs. Clinical data and outcomes were prospectively registered. Genotyping was obtained from a GWAS.

**Results:**

The median 94-SNP risk score in 8703 patients with early breast cancer was 77.5 (range: 58.1–97.6). The risk score was not associated with usual prognostic and predictive factors (age; tumor, node, metastasis (TNM) status; Scarff-Bloom-Richardson grade; inflammatory features; estrogen receptor status; progesterone receptor status; human epidermal growth factor receptor 2 (HER2) status) and did not correlate with breast cancer subtypes. The 94-SNP risk score did not predict outcomes represented by overall survival or disease-free survival.

**Conclusions:**

In a prospective case cohort of 8703 patients, a risk score based on 94 SNPs was not associated with breast cancer characteristics, cancer subtypes, or patients’ outcomes. If we hypothesize that prognosis and subtypes of breast cancer are determined by constitutional genetic factors, our results suggest that a score based on breast cancer risk-associated SNPs is not associated with prognosis.

**Trial registration:**

PHARE cohort: NCT00381901, Sept. 26, 2006 – SIGNAL cohort: INCa RECF1098, Jan. 28, 2009

**Electronic supplementary material:**

The online version of this article (doi:10.1186/s13058-017-0888-4) contains supplementary material, which is available to authorized users.

## Background

Both environmental and genetic factors are involved in breast cancer pathogenesis. Germline mutations in the tumor suppressor genes *BRCA1* and *BRCA2* are the two main genes involved in hereditary breast cancer, and explain around 15–20% of familial breast cancer [1–3]; however, less than 10% of all breast cancers occur in patients with *BRCA* germline mutations [4]. Other rare variants in genes such as *PALB2, CHEK2, ATM, NBN, TP53, CDH1, PTEN, STK11* and *NF1* [5] confer moderate to high risk of developing breast cancer [6]. Genome-wide association studies (GWAS) have to date identified 94 common genetic variants (single nucleotide polymorphisms (SNPs)) associated with risk of developing breast cancer [7]. If the effect of one SNP on breast cancer risk is low, the combined effect of all known associated SNPs can be of interest for prevention and screening, and SNPs explain 15–20% of familial breast cancer [3, 5, 7]. A score based on the effect of risk variants can be calculated to measure the risk of developing breast cancer conferred by the 94 known SNPs [8]. Rare mutations conferring high risk of breast cancer, for example in *BRCA1/2* genes are not included in this score. While SNP scores have been shown to be strongly associated with breast cancer risk, these polygenic SNP scores have not yet been evaluated with respect to clinico-pathological features of breast cancer, prognosis and outcomes.

Clinico-pathological criteria, including patient age, axillary lymph node involvement, tumor size and Scarff-Bloom-Richardson (SBR) grade, are commonly used in the clinical routine as breast cancer prognostic factors; estrogen receptor (ER), progesterone receptor (PR) and human epidermal growth factor receptor 2 (HER2) status are validated as prognostic and predictive factors [9–11]. Based on these predictive factors, medical oncologists divide breast cancers into 3 categories according to the management they require [12, 13]: (1) HER2-positive breast cancers are characterized by amplification of the *HER2* gene (human epidermal growth factor receptor 2, located at 17q12) associated with gene overexpression and consequently high abundance of HER2 protein. The advent of trastuzumab, a humanized monoclonal antibody specifically targeting the HER2 extracellular domain, has revolutionized the natural history and management of HER2-positive breast cancers [14]; (2) triple-negative breast cancer, with no expression of ER or PR and no HER2 overexpression (amplification) has overall poorer prognosis than other subtypes and requires chemotherapy [15]; (3) HER2-negative breast cancers with ER or PR expression represent the third group, called luminal breast cancers, and are usually treated with endocrine therapy [16]. The SIGNAL/*Protocole Herceptin® Adjuvant Réduisant l'Exposition* - Herceptin®-based protocol with reduced exposure (PHARE) - prospective cohort benefits from a large, detailed database allowing assessment of pathological subtypes, prognostic factors and outcomes.

We aimed to test the hypothesis that genetic polymorphisms involved in breast cancer risk may also impact the aggressiveness of breast cancer and thus be related to prognostic factors, pathological subtypes and patients’ outcomes. Individually, genetic variants have a small impact on breast cancer risk, and potentially small consequences on outcomes and pathological features of breast cancer. A polygenic 94-SNP score, which has more statistical power than individual SNPs, may also be associated with breast cancer prognostic factors and outcomes. Our objective was to assess if a polygenic 94-SNP risk score was associated with breast cancer outcomes, prognostic factors and pathological subtypes in the PHARE and SIGNAL French prospective case cohort (NCT00381901 – RECF1098).

## Methods

### Patients

**Fig. 1:**
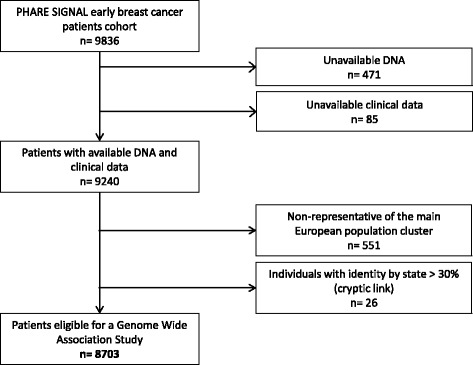
Flow chart

PHARE was a randomized phase III clinical trial comparing 6-month and 12-month adjuvant trastuzumab exposure (NCT00381901) and included a subset of 1430 cases of HER2-positive early breast cancer with DNA available for GWAS analyses [17]. SIGNAL was a prospective cohort specifically designed for GWAS analyses of 8406 patients with early-stage breast cancer, enrolled at the time of their adjuvant chemotherapy from June 2006 to December 2013 (www.e-cancer.fr RECF1098). The combined dataset representing the SIGNAL/PHARE study included 9836 cases of early breast cancer; among them 4834 were HER2-positive breast cancer. All patients provided a blood sample, which was centralized at the Fondation Jean Dausset-Centre d’Etudes du Polymorphisme Humain (CEPH) in Paris, France, for DNA extraction using standard protocols. Genotyping was carried out at the Centre National du Génotypage (CNG) in Evry, France. From the 9836 patients in the SIGNAL/PHARE population, some cases were excluded: 471 patients because there was no DNA available for analysis, among the 26 pairs of individuals with identity by state > 30% (suggesting a cryptic link) the member of the pair with lower genotype completion rate was removed, 551 were non-representative of the main European population cluster, and 85 did not have sufficient clinical data. A total of 8703 patients were analyzed (Fig. 1). Information on patient age, tumors (tumor, node, metastasis (TNM) status, SBR grade, laterality, inflammatory features, ER expression, PR expression and HER2 status) and outcomes (survival, death, breast cancer relapse and second cancer) were prospectively provided directly from the patients’ medical teams using standardized forms, and centralized at the French National Cancer Institute (Institut National du Cancer - INCa).

### Genotyping and 94-SNP risk score

The 94 SNPs used in the risk score were selected based on the literature and were measured in these women as part of a GWAS. These 94 variants are described in the European population [7]. Briefly, all subjects were genotyped using the Illumina HumanCore Exome chip set. Principal components analysis and k-means were then used to characterize the ancestry of the participants and only the main cluster of European individuals was included in the present analysis: 94 SNPs associated with breast cancer risk were selected from recent literature (Additional file 1: Table S1). Sixty-one variants not present in our genotyping arrays were imputed from the 1000 Genomes project (http://1000genomes.org). The cumulative effect of the 94 SNPs was assessed by summing the number of at-risk alleles carried for each individual in an unweighted way. Carrying two low-risk alleles was scored 0; two high-risk alleles were scored 2 and heterozygous status was scored as 1. For imputed SNPs, the estimated allele dose was used directly as the score for each SNP. Thus, the score could range between 0 and 94 × 2 = 188. Supplementary data about subject recruiting, blood collection, DNA extraction, genotyping and imputation are detailed in Additional file 2.

### Statistical analysis

The primary objective was to detect an association between the 94-SNP risk score and invasive-disease-free survival (iDFS) [18]. iDFS was defined as the time from first (neo)adjuvant chemotherapy administration to time of first documented disease relapse (including local, regional, ipsilateral, contralateral and distant invasive breast cancer recurrence), second non-breast malignant disease or death (whatever the cause), whichever occured first [18]. Overall survival (OS) was calculated from the date of diagnosis to the date of death from any cause. For iDFS and OS, patients alive without any predefined event were censored at the time of the last assessment. Survival times were computed according to the Kaplan-Meier method. Results were adjusted for breast cancer type (luminal, HER2 or triple- negative) age at start of treatment, tumor size, nodal involvement and inflammatory type. Breast cancers were divided into three subtypes as defined in the “Background” section: HER2-positive, luminal and triple-negative breast cancers.

The 94-SNP score risk was studied as a continuous variable and subgroups were defined based on quartile values. A relationship was examined between iDFS and OS time and the 94-SNP score risk using Cox proportional hazard models. Differences in mean SNP score and clinical characteristics and between breast cancer subtypes (HER2-positive, luminal and triple-negative) were assessed by analysis of variance (ANOVA). All statistical tests were performed using R version 3.1.2.

A post hoc power analysis using PASS 14 software showed that our study had more than 82% power to detect a hazard ratio (HR) of 1.02 or higher for a change of one unit of the score, considering iDFS and given the sample size of 8703 patients and the observed event rate of 0.118. If we consider a change of 5.48 as the unit, which corresponds to the standard deviation, the HR would then be 1.11.

## Results

### Clinico-pathological characteristics of the population and 94-SNP risk score repartition

**Fig. 2:**
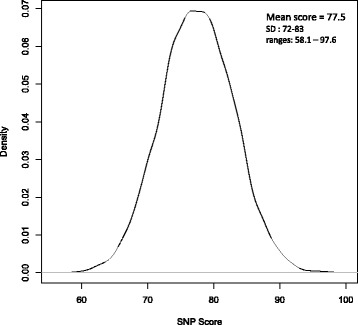
The 94-SNP risk score repartition among the breast cancer patient population: normal distribution. *SNP* single nucleotide polymorphism

From May 30, 2006, to December 30, 2013, 8703 assessable women with early breast cancer were included in the SIGNAL/PHARE cohort (NCT00381901 - RECF1098). The median OS time was 56 months (range 2.7–183, standard deviation +/- 14.5) and the median iDFS time was 54.3 months (range 0–183, standard deviation +/- 16.0). Because of the PHARE study inclusion criteria this cohort was enriched in HER2-positive breast cancer subtypes [17] with 3199 patients (36.8%) with HER2-positive breast cancer. Clinical characteristics are summarized in Table 1. All 94 SNPs were successfully genotyped (33 SNPs) or imputed (61 SNPs). As these SNPs are necessary for calculating the score, no quality filtering was applied to the SNP imputation. The 94-SNP risk median value was 77.5 (range 58.1–97.6) (Fig. 2). The distribution of the risk score among the population was considered as normal.

**Table 1:** Clinico-pathological characteristics of the patients (n = 8703)

Characteristics	Number of patients, or median (range)	Percentage of the study population
Age, years		
median (range)	53.7 (21.8–90.9)	NA
Size of tumor		
T1	4042	46
T2	3416	39
T3–T4	1074	12
missing data	171	2
Nodal status		
N0	4593	53
N1	3052	35
N2	575	7
N3	219	3
missing data	264	3
SBR grade		
I	689	8
II	4034	46
III	3730	43
missing data	250	3
Inflammatory breast cancer		
yes	317	4
no	8201	94
missing data	185	2
Laterality		
right	4197	48
left	4347	50
bilateral	76	1
missing data	83	1
Estrogen receptor status		
negative	2468	28
positive	6191	71
missing data	44	0.5
Progesterone receptor status		
negative	3717	43
positive	4908	56
missing data	78	1
HER2 status		
negative	5504	63
positive	3199	37
Breast cancer subtypes		
triple-negative breast cancer	1115	13
luminal breast cancer	4355	50
HER2-positive breast cancer	3199	37
missing data	34	0.4
Outcomes		
recurrences	1025	12
deaths	423	5
- related to breast cancer	359	4
- unrelated to breast cancer	64	0.7

*SBR* Scarff-Bloom-Richardson, *HER2* human epidermal growth factor receptor 2, *NA* not applicable

### Relationship between SNP risk score and prognosis factors

The 94-SNP risk score was not associated with any of the usual prognosis factors (Table 2). The age at breast cancer diagnosis was not correlated with the 94-SNP risk score (*p* = 0.18). The size of the tumor, the nodal status, the SBR grade and the inflammatory status were not associated with the 94-SNP risk score (*p* > 0.05).

**Table 2:** Association between clinico-pathological characteristics and 94-SNP risk score: no significant correlation

Characteristics	*P* value
Age	0.24
Size of tumor	0.58
Nodal status	0.61
SBR grade	0.89
Inflammatory breast cancer	0.92
Laterality	0.32
ER status	0.77
PR status	0.72
HER2 status	0.49
Breast cancer subtypes	0.79

*SBR* Scarff-Bloom-Richardson, *ER* estrogen receptor, *PR* progesterone receptor, *HER*2 human epidermal growth factor receptor 2

### Predictive factors and breast cancer subtypes

**Fig. 3:**
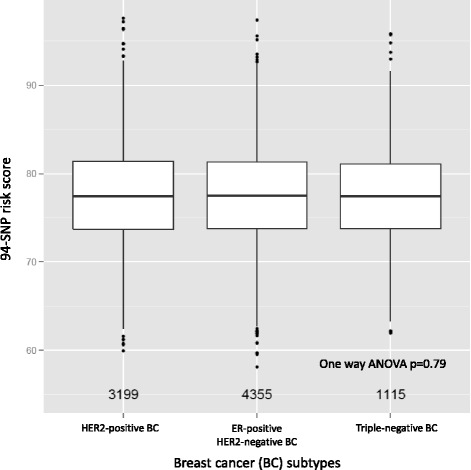
No correlation between the 94-SNP risk score and pathological subtype of breast cancer. *SNP* single nucleotide polymorphism, *ER* estrogen receptor, *HER2* human epidermal growth factor, *ANOVA* analysis of variance

There was no consistent association between the 94-SNP risk score and ER status, PR status or HER2 status (Table 2). The 94-SNP risk score was not correlated with the three clinical subtypes of breast cancer - triple-negative breast cancer, HER2-positive breast cancer and hormone-receptor-positive HER2-negative breast cancer (Fig. 3).

### Outcomes (OS and iDFS)

**Fig. 4:**
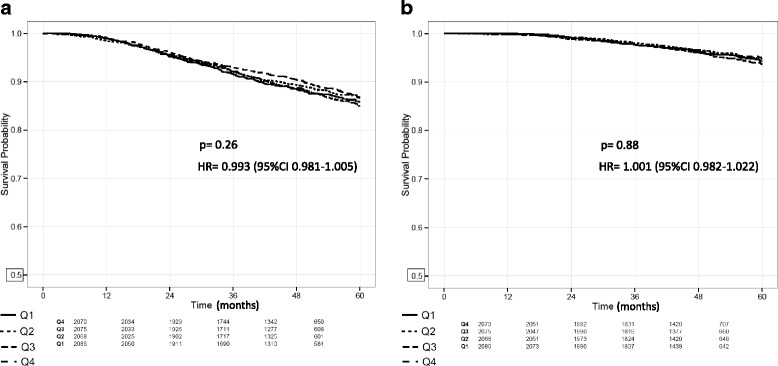
Survival according to 94-SNP risk score quartiles. **a** Disease-free survival. **b** Overall survival. No relationship between invasive-disease-free survival (iDFS) or overall survival (OS) and the 94-SNP risk score. The *p* value and hazard ratio (HR) is from the test of trend from quartile (Q) 1 to quartile 4. *SNP* single nucleotide polymorphism

No relationship was found between survival endpoints and the 94-SNP risk score. No evidence of difference in terms of iDFS or OS between patients in the different quartiles of 94-SNP risk score was observed (Fig. 4); with a *p* value of 0.26 for iDFS at and a HR of 0.993 (95% CI 0.981–1.005). For OS, the *p* value was 0.88 and the HR was 1.001 (95%CI 0.982–1.022).

## Discussion

We have evaluated the prognostic value of a 94-SNP risk score in 8703 patients with early breast cancer included in the PHARE and SIGNAL prospective case cohort (NCT00381901 – RECF1098). This score was not associated with prognostic and predictive factors commonly used in the clinical routine, and was similarly unrelated to breast cancer subtypes. Moreover, the 94-SNP risk score did not predict outcomes. The analysis of this large cohort did not detect any association between iDFS and the 94-SNP score although the study had more than 82% power to detect a HR of 1.02 or higher. A previous GWAS [19] has already suggested that survival may be associated with a different set of SNPs to those that influence breast cancer susceptibility. If we hypothesize that prognosis and subtype of breast cancer are determined by constitutional genetic factors, variants associated with breast cancer subtypes and prognosis may be different from variants involved in the risk of developing breast cancer. Tumoral characteristics and age at diagnosis were superimposable between patients at high and low risk. Even if we assume that patients with family history of breast cancer may have a higher genetic risk score, breast cancer characteristics and outcomes of these high-risk patients are similar to others. Genetic history has already provided such an example: *BRCA1* and *BRCA2* gene mutations significantly increase the risk of developing breast cancer; however, outcomes of carriers seem to be similar to those with sporadic breast cancer [20–26]. For each individual, we calculated a 94-SNP score by adding the number of breast cancer risk-increasing alleles across 94 known breast cancer SNPs. All variants are equally weighted. *BRCA1/2* variants, which are rare and confer high risk of cancer, are not included in the 94-SNP score. Risk scores are generally calculated this way [3, 7]; however, these points can be considered as limits. Furthermore, we did not apply any quality filtering for imputed SNPs. There may be very minor error in calculating the overall risk score when including poorly imputed SNPs, but this impact should be minor considering the number of SNPs involved.

The first studies for identification of variants associated with prognosis in breast cancer investigated polymorphisms of candidate genes involved in oncogenesis, such as *Plasminogen activator inhibitor-1 gene* [27, 28], *VEGF* [29], *TP53* [30] or *Cycline D1* genes [31] and suggested links between some gene variants and breast cancer prognosis. Recently, GWAS have focused on associations between inherited germline genetic variants and breast cancer outcomes. They have identified SNPs that may influence breast cancer prognosis [28, 32–34]. Around 60 variants have been described to date as potentially correlated with breast cancer outcomes [35]. Most of them are involved in pathways playing fundamental roles in oncogenesis such as cell cycle control, cell adhesion or DNA repair [35, 36]. However, in a cohort of over 37,000 patients with breast cancer, none of the 62 studied variants showed significant association with outcomes [35, 37–42]. From these 62 variants, only one (rs2981582, in *FGFR2* on chromosome 10) is used in our 94-SNP score. It has been identified as possibly associated with outcomes in breast cancer, with a HR (90% CI) of 1.09 (1.04–1.14) [35]. This variant reached nominal significance (*p* < 0.05) but did not reach genome-wide significance (*p* < 5 × 10^−8^) [35]. Preliminary analyses in our GWAS study do not indicate that this variant is associated with outcomes (unpublished data). This lack of evidence can be explained by limited statistical power, or that germline genetic polymorphisms may not impact the natural history of breast cancer, once the cancer is present.

Regarding breast cancer subtype, there is more evidence that susceptibility loci are associated with specific breast cancer subtypes. In 2011, the Breast Cancer Association Consortium identified six loci associated with ER+ breast cancer, four loci associated with triple-negative tumors and two loci associated with basal-like tumors [43]. These variants were included in the present analyses. The SIGNAL/PHARE cohort confirmed the association between *FGFR2* locus and ER+ tumors, further restricting this association with HER2-negative breast tumors [44]. In our study, the 94-SNP risk score was not associated with specific breast cancer subtypes.

In clinical practice, there is a need to identify prognostic factors that can predict the risk of tumor recurrence. To accurately determine the prognosis of a patient is crucial and can also help to stratify patients in clinical trials assessing new therapies. Finding predictive factors that are associated with response or failure to a treatment and thus help to identify the most effective therapy remains the ultimate challenge to provide patients with personalized medicine. With regard to this aim, gene expression signatures assessed on tumor tissue, such as the 21-gene recurrence score assay Oncotype DX®, Mammaprint®, EndoPredict® or PAM50®, are of interest. They estimate the risk of distant recurrence and Oncotype DX® also predicts the magnitude of benefit of adjuvant chemotherapy for patients with early-stage breast cancer [45–49]. Genes involved in this signature are different from those used in the 94-SNP score. Genetic variants and scores based on SNPs may be of interest in clinical routine if they provide prognostic and predictive information [50, 51]. GWAS in very large case cohorts of patients with available complete clinical data provide the opportunity to identify prognostic and predictive variants usable in clinical practice. The SIGNAL/PHARE database will also allow the investigation of clinical endpoints such as iDFS. The SIGNAL trial is the first large prospective clinical study whose primary objective was to identify prognostic and predictive genetic variants in early breast cancer. We are currently expanding our analyses, in order to search for SNPs associated with prognostic and predictive factors, eventually combined in a polygenic risk score as described for breast cancer risk, which could be of interest in routine clinical practice. Further stratifying patients based on their potential to respond to treatment will help optimize adjuvant regimens, if indeed they are necessary.

## Conclusion

A score built with 94 SNPs can be used to stratify women with respect to their risk of developing breast cancer. In a prospective cohort of 8703 patients, this score is not associated with breast cancer characteristics, cancer subtypes or patients’ outcomes (iDFS and OS). If we hypothesize that prognosis and subtypes of breast cancer are determined by constitutional genetic factors, we suggest that inherited variants associated with breast cancer subtypes and prognosis may be different from variants involved in the risk of developing breast cancer.

## Additional files

Additional file 1: **Table S1.** List of 94 variants associated with risk, involved in the 94-SNP risk score (identification and characteristics of variants). (XLS 41 kb)

Additional file 2: Supplementary methods. supplementary data on subject recruiting, blood collection, DNA extraction, genotyping and imputation. (DOCX 26 kb)

## Abbreviations

ANOVA: Analysis of variance
ER: Estrogen receptor
GWAS: Genome-wide association study
HER2: Human epidermal growth factor receptor 2
HR: Hazard ratio
iDFS: Invasive disease-free survival
OS: Overall survival
PR: Progesterone receptor
SBR: Scarff-Bloom-Richardson
SNP(s): Single nucleotide polymorphism(s)
TNM: Tumor, node, metastasis
