# Supplementary material for: Assessment of the prognostic role of a 94-single nucleotide polymorphisms risk score in early breast cancer in the SIGNAL/PHARE prospective cohort: no correlation with clinico-pathological characteristics and outcomes
Source: Breast Cancer Res. 2017 Aug 22;19:98. doi: 10.1186/s13058-017-0888-4 (PMC5568360; doi:10.1186/s13058-017-0888-4)
Supplement: Supplementary file 2 — Supplementary methods. supplementary data on subject recruiting, blood collection, DNA extraction, genotyping and imputation. (DOCX 26 kb) [file 13058_2017_888_MOESM2_ESM.docx]

**Assessment of the prognostic role of a 94-Single Nucleotide Polymorphisms risk score in early breast cancer in the SIGNAL/PHARE prospective cohort: no correlation with clinico-pathological characteristics and outcomes**

Elsa Curtit^1^*, Xavier Pivot^1^, Julie Henriques^2^, Sophie Paget-Bailly^2^, Pierre Fumoleau^3^, Maria Rios^4^, Hervé Bonnefoi^5^, Thomas Bachelot^6^, Patrick Soulié^7^, Christelle Jouannaud^8^, Hugues Bourgeois^9^, Thierry Petit^10^, Isabelle Tennevet^11^, David Assouline^12^, Marie-Christine Mathieu^13^, Jean-Philippe Jacquin^14^, Sandrine Lavau-Denes^15^, Ariane Darut-Jouve^16^, Jean-Marc Ferrero^17^, Carole Tarpin^18^, Christelle Lévy^19^, Valérie Delecroix^20^, Véronique Trillet-Lenoir^21^, Oana Cojocarasu^22^, Jérôme Meunier^23^, Jean-Yves Pierga^24^, Pierre Kerbrat^25^, Céline Faure-Mercier^26^, Hélène Blanché^27^, Mourad Sahbatou^27^, Anne Boland^28^, Delphine Bacq^28^, Céline Besse^28^, Gilles Thomas^29^, Jean-François Deleuze^27-28^, Iris Pauporté^26^, Gilles Romieu^30^, David Cox^31^

**Supplementary methods**

**Subject recruiting, blood collection and DNA extraction**

Eligibility criteria included the following: patients over 18 years of age, with histologically confirmed invasive breast cancer. Patients must have received (neo)adjuvant chemotherapy and/or breast-axillary surgery before recruitment, and signed informed consent. HER2 status was determined by a certified local laboratory using immunohistochemistry or fluorescence *in situ* hybridization. Other clinical characteristics were determined from pathology reports. Blood samples were collected at the time of inclusion in the study, after the patient signed the informed consent. All patients signed full informed consent. Blood samples were collected on EDTA and Citrate, and shipped via courier at 4°C to the CEPH. Plasma and buffy-coat were isolated from EDTA after centrifugation at 1600g for 10 min at 4°C. Lymphocytes isolated from citrate blood using a density gradient centrifugation method on “UNI-SEP Lymphocyte Separation” tubes (Eurobio, Courtaboeuf, France) and following manufacturer’s instructions. DNA was extracted from buffy-coat using salting out protocols on the Autopure LS (Qiagen, Venlo, Netherlands) provided by the manufacturer. DNA concentrations were measured using “PicoGreen dsDNA reagent” (Life Technologies, USA). DNAs were diluted sequentially using TE 10:1 to obtain concentrations normalized at 100ng/μl. DNA and plasma samples are then stored at -80°C, while viable lymphocytes are stored in liquid nitrogen.

**Genotyping and imputation**

All subjects were genotyped using the Illumina HumanCore Exome chip set. SNPs with >5% missing data, a Hardy-Weinberg p-value < 0.001, or a minor allele frequency <0.1%, and that were present in duplicate or triplicate were excluded from further analysis. Principal Components Analysis and k-means were then used to characterize the ancestry of the participants. These analyses were conducted using the EIGENSTRAT program of the smartpca.perl package, followed by k-means clustering using the kmeans function of the NbClust package in R. Hapmap subjects were included in order to provide scale and points of reference for European, African, and Asian clusters. Only the main cluster of European individuals was included in the present analysis. These data were then used to impute missing genotypes for SNPs needed to generate the polygenic risk score, using variants in the linkage disequilibrium block around the imputed SNP (R^2^>0.80). The 1000 Genomes project (<http://1000genomes.org>) data were used as the reference, and HumanCore Exome data were imputed to this reference using Minimac3.
